# Recent advances in diffusion neuroimaging: applications in the developing preterm brain

**DOI:** 10.12688/f1000research.15073.1

**Published:** 2018-08-21

**Authors:** Diliana Pecheva, Christopher Kelly, Jessica Kimpton, Alexandra Bonthrone, Dafnis Batalle, Hui Zhang, Serena J. Counsell

**Affiliations:** 1 Centre for the Developing Brain, School of Biomedical Engineering & Imaging Sciences, King's College London, London, UK; 2 Department of Computer Science & Centre for Medical Image Computing, University College London, London, UK

**Keywords:** infant, brain, diffusion magnetic resonance imaging

## Abstract

Measures obtained from diffusion-weighted imaging provide objective indices of white matter development and injury in the developing preterm brain. To date, diffusion tensor imaging (DTI) has been used widely, highlighting differences in fractional anisotropy (FA) and mean diffusivity (MD) between preterm infants at term and healthy term controls; altered white matter development associated with a number of perinatal risk factors; and correlations between FA values in the white matter in the neonatal period and subsequent neurodevelopmental outcome. Recent developments, including neurite orientation dispersion and density imaging (NODDI) and fixel-based analysis (FBA), enable white matter microstructure to be assessed in detail. Constrained spherical deconvolution (CSD) enables multiple fibre populations in an imaging voxel to be resolved and allows delineation of fibres that traverse regions of fibre-crossings, such as the arcuate fasciculus and cerebellar–cortical pathways. This review summarises DTI findings in the preterm brain and discusses initial findings in this population using CSD, NODDI, and FBA.

## Introduction

Diffusion-weighted magnetic resonance imaging (dMRI) is a non-invasive imaging technique that measures the displacement of water molecules in tissue over time. As such, dMRI offers the opportunity to investigate tissue microstructure *in vivo* and provides quantitative measures that relate to brain injury and development. The most widely used dMRI analysis approach in the developing brain is diffusion tensor imaging (DTI), which has proven to be extremely useful for investigating brain development and injury. More recent approaches have moved beyond the tensor model to incorporate biophysical models to study tissue microstructure more specifically.

The aim of this review is to briefly describe studies assessing white and grey matter in the developing brain using advanced analysis approaches that have been used widely in the adult brain, such as neurite orientation dispersion and density imaging (NODDI), fixel-based analysis (FBA), and constrained spherical deconvolution (CSD). These approaches require high b-value (typically >2,000 s/mm ^2^), high angular resolution dMRI data which pose additional challenges in neonatal imaging including longer acquisition times, which may lead to motion corrupt data, reduced signal-to-noise ratio, and increased distortions. However, recent advances in data acquisition approaches and hardware, coupled with imaging at 3 Tesla, mean it is now possible to acquire high-quality HARDI data in the neonatal brain ^1^. We believe these techniques will be increasingly used to improve our understanding of the neural substrate associated with impaired brain development in this population.

## Diffusion-weighted imaging

Diffusion is the constant motion of molecules due to thermal energy. Given an environment without restrictions, water molecules will traverse a random walk, with direction changes following collisions with other particles. However, in the brain, the presence of axons, neuronal cell bodies, glial cells, and macromolecules comprise a heterogeneous environment which hinders and restricts diffusion. In the presence of these impediments, the measured root-mean-square displacement will be lower than predicted for water at room temperature. The term “apparent diffusion coefficient” is used to convey that the observed measure is influenced by the tissue microstructure. Diffusion is restricted if it is confined by physical boundaries, such as the diffusion of molecules in the intra-axonal space, causing the diffusion to become non-Gaussian ^2, 3^.

## Diffusion tensor imaging

**Figure 1.**
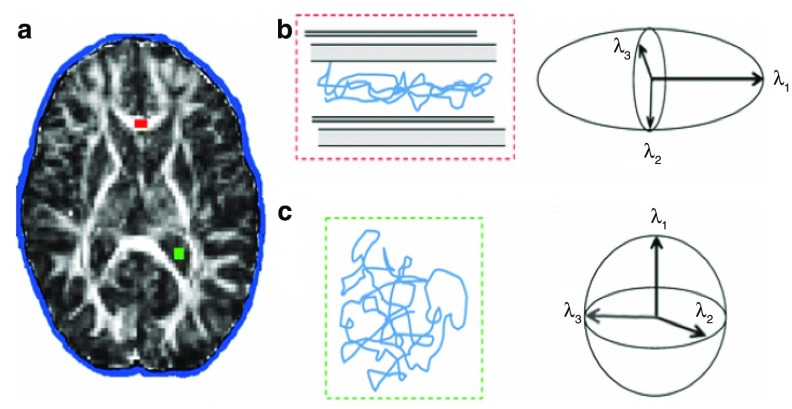
Isotropic and anisotropic diffusion in the brain. In the white matter of the corpus callosum (red), diffusion occurs preferentially along the axonal fibres, resulting in anisotropic diffusion ( **b**). In the ventricular cerebrospinal fluid (CSF; green), diffusion is unhindered and can be described as isotropic ( **c**). Diffusion tensor ellipsoids representing anisotropic and isotropic diffusion are shown in **b** and **c**, respectively. Reproduced with permission from 9.

The organisation of tissue microstructure will affect how water molecules diffuse. In a homogenous medium, the diffusion of water molecules is equal in all directions; this is isotropic diffusion. However, in a coherently organised microstructure, such as in white matter, diffusion is anisotropic ^4– 6^. Within white matter, water molecules diffuse more slowly perpendicular to the fibres than parallel to them ( Figure 1). Under these conditions, the apparent diffusion coefficient will be different depending on the direction in which it is measured. To account for this, Basser *et al*. ^7^ proposed that diffusion is characterised using a mathematical tensor model. To examine water molecular motion in a tissue with an ordered microstructure using the tensor model, a minimum of six non-collinear directions of diffusion sensitisation is required, in addition to one with no diffusion weighting, although usually at least 30 unique sensitised directions are recommended to robustly estimate the tensor model parameters.

The diffusion tensor provides scalar, rotationally invariant indices ^8^. Indices derived from λ _1_, λ _2_, λ _3_ ( Figure 1) are, by definition, independent of orientation. The magnitude of the diffusivity along the main fibre orientation as estimated by DTI is given by λ _1_, termed the axial diffusivity (AD). The average of the other two eigenvalues, the radial diffusivity (RD), describes the magnitude of diffusivity across the fibres. The mean diffusivity (MD) is the average of all three eigenvalues and provides a measure of the overall diffusivity within a voxel. Fractional anisotropy (FA) is the variance of the three eigenvalues normalised by the magnitude of the tensor and takes values between 0 and 1.

## DTI studies in the infant brain

The perinatal period is characterised by a pattern of decreasing MD, RD, and AD and increasing FA in the cerebral white matter in preterm infants ^10– 14^ and term infants ^15, 16^. White matter maturation follows a heterogeneous spatiotemporal pattern, with different fasciculi maturing at different times and different rates ^15– 23^ in a posterior-to-anterior and a central-to-peripheral direction of maturation. The increase in FA takes place before myelin is evident histologically and is attributed to changes in white matter structure which accompany the premyelinating state including an increase in axonal membrane maturation and microtubule-associated proteins, a change in axon caliber, and an increase in oligodendrocyte number ^24– 26^. At this stage, the highest FA values are seen in the unmyelinated but highly organised commissural fibres in the splenium and genu of the corpus callosum. The second stage is associated with the histological appearance of myelin and subsequent maturation, with the earliest signs observed in the projection fibres of the posterior limb of the internal capsule around term ^27^.

Lower FA and increased MD are found across the white matter in preterm infants compared with term-born infants ^24, 28– 30^, and increased prematurity is associated with lower FA and higher MD ^13, 31– 35^. Furthermore, infants with white matter injury identified on conventional MRI show reduced anisotropy and increased MD and RD across the white matter in comparison to preterm infants with normal MRI ^14, 36– 40^. White matter diffusion measures in preterm infants at term equivalent age have been related to subsequent neurodevelopmental performance. Increased FA and decreased MD and RD in the white matter at term equivalent age are associated with improved motor, cognitive, and language performance in early childhood ^41– 47^ and improved visual function ^48– 50^.

In addition to assessing white matter, DTI studies of cortical grey matter have identified altered cortical development in infants born preterm. Cortical maturation up to term equivalent age is characterised by decreasing FA and MD, reflecting increased dendritic arborisation and synapse formation ^35, 51– 53^. FA and MD are elevated in preterm infants at term equivalent age compared to infants born at term, suggesting impaired cortical development in this population ^52^.

## Limitations of DTI

While DTI has proven to be a powerful technique for studying the brain, a major limitation is that it is only able to depict a single fibre population within a voxel. DTI fails to represent appropriately the tissue microstructure in the presence of crossing fibres and DTI-derived measures lack tissue specificity, as these measures can be affected by multiple microstructural features. Moreover, in a restricted environment, diffusion is no longer Gaussian and the tensor model deviates from the signal. The use of more advanced analysis approaches, such as those that enable microstructure to be studied with greater specificity, require high angular resolution diffusion imaging (HARDI) acquisitions at a higher b-value than has typically been used in the neonatal brain. These approaches have long acquisition times and so their use in unsedated neonates has been limited. However, advances in MRI acquisition techniques, such as the use of protocols designed specifically for neonates using neonatal head coils and multiband MRI coupled with modern gradient coil systems, with maximum gradient amplitude, slew rate, and duty cycle ^1, 54^, now enable HARDI data to be acquired in a clinically feasible time.

## Compartment models of microstructure

Compartment models provide a biophysical interpretation of the diffusion-weighted signal and attempt to characterise the complexity of cerebral tissue by decomposing the signal into compartments describing diffusion within distinct microstructural constituents.

Stanisz *et al*. ^55^ first introduced the three-compartment model comprising a restricted intra-axonal compartment, anisotropic hindered extra-axonal compartment, and a restricted isotropic compartment describing diffusion within cellular structures such as glial cells. Behrens *et al*. ^56^ presented a method to account for multiple fibre populations using the ball and stick model where diffusion along axons is represented by sticks and outside the axons diffusion is an isotropic ball. CHARMED ^57^ models the intra-axonal space using cylinders with a distribution of radii given by the Γ-distribution and extra-axonal space as tensor with a principle direction aligned with the cylinders. This was extended to provide an estimate of axon diameter in the AxCaliber framework ^58, 59^. Alexander ^60^ simplified CHARMED by using a single axon radius and symmetric tensor that was used in the ActiveAx framework to estimate axon diameter in biological tissue ^61, 62^ and axon diameter mapping in the presence of orientation dispersion ^63^. However, recent work shows that the gradient amplitudes attainable with current clinical scanners are not able to estimate axon diameter accurately ^64, 65^.

NODDI ^66^ provides measures of neurite density index (NDI) and orientation dispersion index (ODI). The model consists of three compartments modelling the intracellular, extracellular, and cerebrospinal fluid (CSF) environments. The intraneurite compartment captures the diffusion inside dendrites and axons, collectively termed neurites. The intraneurite compartment is modelled using sticks to represent unhindered diffusion along the neurites and highly restricted diffusion perpendicular to the neurites. The orientation distribution can vary from being highly parallel, reflecting the coherent organisation of white matter fibres such as in the posterior limb of the internal capsule or the corpus callosum, to highly dispersed, such as in regions of crossing fibres like the centrum semiovale or the complex configuration of the cortex. The extraneurite compartment represents the space occupied by glial cells and neuronal somas where diffusion is hindered and is modelled as an anisotropic Gaussian distribution using a zeppelin. The CSF compartment is modelled as isotropic Gaussian diffusion.

**Figure 2.**
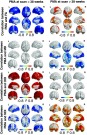
Correlation between cortical diffusion characteristics and age at scan. Hot colours indicate increase and cool colours indicate decrease in diffusion measure. Abbreviations: FA, fractional anisotropy; MD, mean diffusivity; NDI, neurite density index; ODI, orientation dispersion index; PMA, post-menstrual age. Reproduced from 34.

The NODDI model has been applied to investigate white and grey matter maturation in the preterm brain ^34, 67, 68^. NDI increases in the white matter with increasing maturation, with the highest NDI values observed in primary motor and somatosensory tracts and lower values observed in association fibres ^67, 69^. Combined with graph theoretical approaches and network-based analysis, both FA- and NDI-weighted connections were highly correlated with age at MRI in a widespread pattern encompassing most white matter connections between 25 and 45 weeks post-menstrual age (PMA). Lower gestational age (GA) at birth was significantly correlated with lower FA and NDI, and we observed a consistent negative correlation of relative NDI-weighted global efficiency with GA at birth, suggesting an alteration in network topology with increased prematurity at birth ^67^. Cortical grey matter maturation is characterised by increasing ODI (accompanied by decreasing MD and FA), reflecting increased dendritic arborisation. At around 38 weeks’ GA, this increase in ODI plateaued, but after this period NDI increased in primary motor and sensory regions ( Figure 2), suggesting that cortical development up to 38 weeks’ PMA shows a predominant increase in dendritic arborisation and neurite growth, while after 38 weeks’ PMA it is dominated by increasing cellular and organelle density ^34^.

The DIAMOND model ^70^ combines compartmental and statistical modelling to represent restricted, hindered, and isotropic compartments using three peak-shaped matrix-variate distributions. DIAMOND estimates the number of tissue compartments in each voxel and provides compartment-specific measures of FA, AD, RD, and MD and a measure of heterogeneity within the compartment. This model was recently applied to assess cortical maturation in the preterm cortex, demonstrating a decrease in the radial organisation of the cortex ^71^.

Approaches to model the diffusion signal have limitations, including assuming non-exchanging tissue compartments and fixed compartmental diffusivities ^72^, and, to date, there have been no studies validating these measures with human preterm or neonatal tissue samples. However, ODI measures have recently been correlated with changes in neurite geometrical configuration assessed with histology in a population with spinal cord multiple sclerosis ^73^, suggesting that model indices are relevant proxies of underlying microstructure.

## Constrained spherical deconvolution

CSD estimates the fibre orientation distribution (FOD) in the presence of multiple fibre orientations ^74– 78^. It was initially introduced for single-shell HARDI data ^79^ and is able to estimate FODs regardless of the number of fibre populations within a voxel. It is assumed that each fibre bundle has the same diffusion properties, apart from the orientation, and that no exchange occurs between bundles over the time-scale of DWI acquisition. The signal emanating from each fibre bundle is independent and they can be summed. The diffusion-attenuated profile for an anisotropic fibre bundle is represented by a response function. The response function is low amplitude along the axis, where diffusion is high, and high amplitude in the radial plane, where diffusion is low. Recently, multi-tissue CSD has been introduced, which exploits the different diffusion dependencies of different tissues at multiple b-values (b-value refers to the degree of diffusion weighting which is related to the amplitude and duration of the diffusion gradients and the time interval between the leading edges of the two pulsed gradients) to derive tissue-specific response functions, where grey matter and CSF response functions are both isotropic, leading to improved estimation of the FOD ^80^.

**Figure 3.**
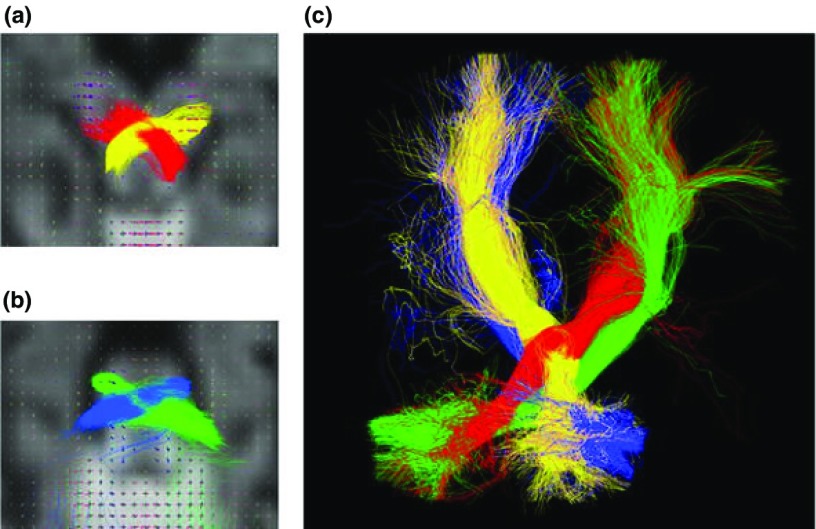
Reconstruction of cerebello–thalamo–cortical tract (CTC, red-yellow) and cortico–ponto–cerebellar tract (CPC, blue-green) in an infant born at 33 weeks and imaged at 40 weeks post-menstrual age with fibre orientation distribution plots overlaid on the diffusion data. ( **a**) Crossing fibres of the CTC tract at the level of the mesencephalon. ( **b**) Crossing fibres of the CPC tract at the level of the pons. ( **c**) 3D reconstruction of both tracts. Reproduced from 81.

**Figure 4.**
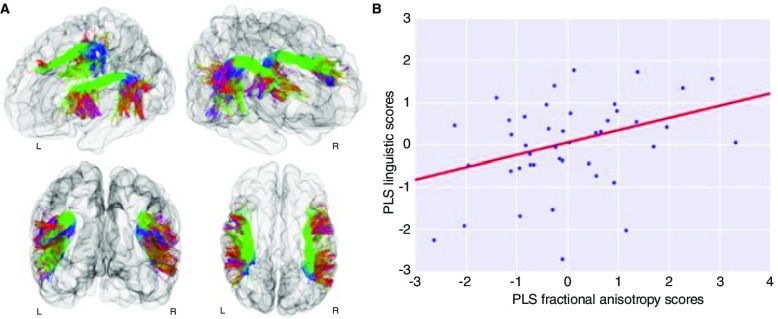
Inter-subject differences in linguistic performance at two years were associated with term equivalent fractional anisotropy (FA) of the left and right arcuate fasciculus independently of degree of prematurity. ( **a**) Visualisation of an infant brain and the reconstructed arcuate fasciculi from left-frontal, right-frontal, frontal, and top view. The tracts are coloured by direction: green for anterior-posterior, red for left-right, and blue for superior-inferior. ( **b**) Using cross-validated partial-least-square (PLS) regression, one statistically significant mode of brain-behaviour covariation between PLS FA scores and PLS language scores was identified ( *r* = 0.36; family-wise error [FWE]-corrected *P*-value = 0.0110). Term equivalent FA of the left and right arcuate fasciculi was associated with individual differences in composite linguistic skills in early childhood. This link was still present even when controlling for degree of premature delivery measured by gestational age (GA) at birth ( *r* = 0.32, FWE-corrected *P*-value = 0.0230). Reproduced from 82.

CSD-based tractography has been used in a limited number of studies in the infant brain to visualise fibre bundles that are not readily delineated using DTI-based approaches. In Pieterman *et al*. ^81^, we were able to visualise cerebellar–cortical pathways crossing in the mid-brain ( Figure 3). In another recent study, we were able to delineate the arcuate fasciculus, which traverses regions of fibre crossings in the centrum semiovale and we observed that FA values of the arcuate fasciculus in preterm infants at term equivalent age correlated with language performance at 2 years ( Figure 4) ^82^.

## Fixel-based analysis

**Figure 5.**
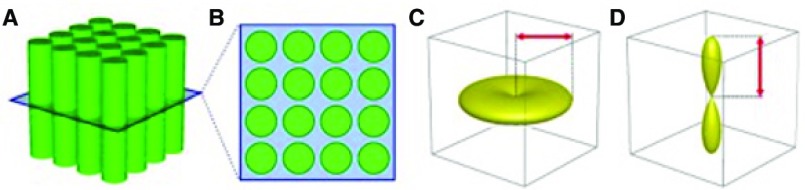
A single fibre population within a voxel ( **A**, **B**), the expected diffusion-weighted signal profile ( **C**), and the associated fibre orientation distribution (FOD) ( **D**) The FOD amplitude is proportional to the radial signal profile and therefore the fibre density of the fibre population. Image adapted from 88.

CSD has led to the development of fibre bundle-specific measures. Raffelt *et al*. ^83^ introduced a measure of apparent fibre density (AFD) of individual fibre populations estimated from the FOD. A fixel describes an individual different fibre bundle within an imaging voxel where fibre bundles of different orientations may be present in an imaging voxel. AFD is based on the assumptions that the intra-axonal water diffusion is restricted in the direction perpendicular to the fibre orientation, the extra-axonal diffusion-weighted signal is attenuated at high b-values (>2,000 s/mm ^2^), and the diffusion-weighted signal from the restricted compartment is preserved under typical diffusion-weighted gradient pulse durations used *in vivo*. Consequently, the radial diffusion-weighted signal is approximately proportional to the volume of the intra-axonal compartment ^83^. Since the FOD amplitude is proportional to the radial diffusion-weighted signal, it provides a measure of fibre density (FD) determined as a proportion of the volume occupied by the fibre population ^83^, as illustrated by Figure 5. This measure would detect within-voxel changes related to the volume of restricted water along a specific direction. AFD also accounts for differences in macroscopic white matter structure across subjects. FODs are modulated according to changes in local volume, such as expansion or contraction, that occur during registration. This presents a measure pertaining to both microscopic changes in FD and macroscopic morphological changes that occur across voxels.

**Figure 6.**
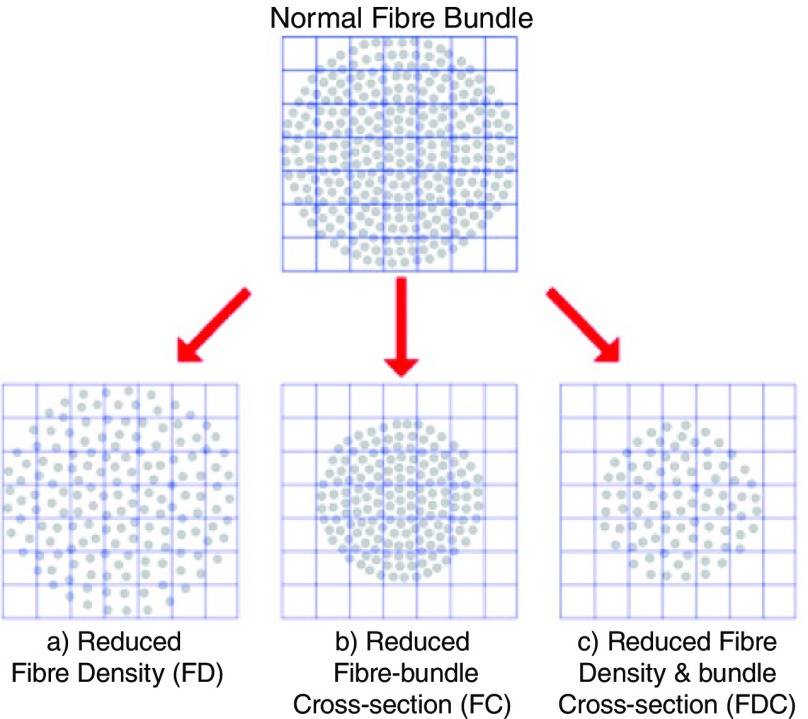
A schematic representation of a fibre bundle cross-section made of numerous axons (grey circles). Anterior commissure voxels represented by the grid. Panels ( **a**), ( **b**), and ( **c**) describe three different ways in which a fibre bundle can change: ( **a**) a reduction in within-voxel fibre density, ( **b**) a macroscopic change in fibre-cross section across voxels, and ( **c**) a combination of reductions in both fibre density and cross-section. Image adapted from 84.

Raffelt *et al*. ^84^ make a distinction between the changes in microstructure that occur within a voxel and the macroscopic changes in morphology that occur across voxels. They introduced a measure of FD derived solely from unmodulated FOD amplitude so as to describe changes in white matter microstructure without the effects of macroscopic morphological changes. Changes in white matter microstructure which would result in a reduction in FD can be visualised in Figure 6. Nonetheless, macroscopic alterations in morphology are likely to occur across white matter during development and need to be accounted for. Raffelt *et al*. ^84^ provide, in addition to FD, a measure of macroscopic differences in morphology based on the local deformations that are applied during registration. Changes in brain morphology have previously been investigated using voxel-based morphometry (VBM) ^85^ and tensor-based morphometry ^86, 87^. Local changes in volume can be investigated using the information from a subject's nonlinear deformation to a template. At each voxel, the determinant of the Jacobian describes the expansion or contraction of the subject image relative to a target. This method focuses on the changes in fibre bundle that occur perpendicular to the main fibre orientation, as a reduced fibre bundle cross-section would imply a reduced number of axons. Using FOD registration, it is possible to assess changes in volume with respect to specific fibre orientations. This provides a fibre bundle-specific measure of fibre cross-section (FC) based on the Jacobian determinant following registration of FOD images.

To date, there have been few studies assessing white matter in the preterm brain using FBA. Pannek *et al*. demonstrated reduced FD, FC, and FD multiplied by FC (FDC) in the corticospinal tract and corpus callosum in preterm infants at term equivalent age compared to healthy controls ^89^. We have observed a significant negative correlation between FC and FDC and duration of mechanical ventilation and parenteral nutrition in preterm infants at term equivalent age, suggesting that aberrant white matter development previously attributed to microstructural changes may be due to alterations in the size (fibre cross-sectional area) of specific fibre bundles at the macroscopic scale ^90^.

## Summary

Recent advances in diffusion acquisition and analysis approaches enable white and grey matter microstructure to be probed in detail, demonstrating increases in NDI and FC in white matter and increasing ODI in cortical grey matter with increasing maturation. CSD-based tractography facilitates the delineation of complex fibre bundles that have not been clearly depicted using DTI approaches. Large-scale studies (such as the developing Human Connectome Project, http://www.developingconnectome.org) are now underway and are obtaining high b-value HARDI data in the neonatal brain with the aim of improving our understanding of human brain development and the impact of environmental and genetic factors on brain development. It is likely that the acquisition and analysis techniques outlined in this review will be confined to the research environment in the short term. However, the ultimate aim of neonatal neuroimaging is to facilitate early diagnosis and prognosis, and innovations in image acquisition including multiband techniques to reduce acquisition time are likely to facilitate the increased use of these advanced methods in the neonatal brain in the future.
